# A multicenter study on preoperative WHO/ISUP grading of clear cell renal cell carcinoma using triphasic contrast-enhanced CT-based habitat imaging

**DOI:** 10.1186/s12880-026-02236-z

**Published:** 2026-02-27

**Authors:** Lei Zhang, Nian Shi, Xiaoyu Chen, Songan Shang, Siyuan Lu, Tianyu Li, Yong Liu, Lei Han, Jing Ye

**Affiliations:** 1 Graduate School of Dalian Medical University, Dalian Medical University, Dalian, Liaoning China; 2 Department of Radiology, Huai’an Second People’s Hospital, The Affiliated Huaian Hospital of Xuzhou Medical University, Huaian, Jiangsu China; 3 Department of Radiology, The Yangzhou Clinical Medical College of Xuzhou Medical University, Yangzhou, Jiangsu China; 4 Department of Radiology, Northern Jiangsu People’s Hospital, Yangzhou, Jiangsu China; 5 Department of Radiology, Northern Jiangsu People’s Hospital, Yangzhou University, Northern Jiangsu People’s Hospital, Yangzhou, Jiangsu China

**Keywords:** Clear cell renal cell carcinoma, Triphasic contrast-enhanced CT, WHO/ISUP grade, Habitat, K-means clustering

## Abstract

**Objective:**

This study aimed to develop a triphasic contrast-enhanced CT-based habitat imaging method for preoperative prediction of World Health Organization/International Society of Urological Pathology (WHO/ISUP) grade in clear cell renal cell carcinoma (ccRCC).

**Methods:**

A retrospective analysis included 300 ccRCC patients from two centers. Center 1 data were used for training (*n* = 190) and internal validation (*n* = 82), and Center 2 for external validation (*n* = 28). All patients underwent triphasic CT scans. Tumor volumes of interest (VOIs) were manually delineated using 3D Slicer. CT values from the corticomedullary (CMP), nephrographic (NP), and excretory (EP) phases were extracted to assess enhancement. K-means clustering segmented tumors into four habitats, and volume fractions were calculated. Logistic regression identified significant predictors from habitat features and clinical variables. A nomogram was constructed and evaluated using receiver operating characteristic (ROC) curves, area under the curve (AUC), calibration curves, Hosmer-Lemeshow (HL) tests, and decision curve analysis (DCA).

**Results:**

Gender, tumor size, and the volume fractions of Habitat 1 (F1) and Habitat 2 (F2) were independent predictors. These predictors were integrated into a nomogram that achieved AUCs of 0.794 (95% CI, 0.726–0.862) in the training cohort, 0.787 (95% CI, 0.678–0.897) in the internal validation cohort, and 0.781 (95% CI, 0.599–0.962) in the external validation cohort. The model showed acceptable calibration and yielded potential net clinical benefit in both validation sets.

**Conclusion:**

We developed and externally validated a CT-based nomogram for preoperative WHO/ISUP grade stratification in ccRCC; larger independent cohorts are needed to confirm generalizability.

**Supplementary Information:**

The online version contains supplementary material available at 10.1186/s12880-026-02236-z.

## Introduction

Clear cell renal cell carcinoma (ccRCC) is the most common malignant kidney tumor, accounting for 70% to 90% of all renal cancers [1]. This disease is typically characterized by high invasiveness and a strong tendency to metastasize, leading to a poor prognosis for affected patients [2]. The clinical management of ccRCC heavily relies on tumor staging and grading, with grading playing a crucial role in prognostic assessment and treatment decision-making. The WHO/ISUP (World Health Organization/International Society of Urological Pathology) grading system is the most widely adopted standard for ccRCC grading, categorizing tumors into four grades based on histological features. The higher the grade, the greater the malignancy and metastatic potential [3, 4]. Pathological grading is typically performed on surgical specimens, while preoperative assessment often depends on invasive procedures such as needle biopsy. Although biopsy is the conventional method for tumor grading, it may fail to fully reflect the pathological characteristics of the tumor due to spatial and temporal heterogeneity [5], potentially leading to misdiagnosis or misclassification [6].

Habitat imaging, as a non-invasive assessment technique, provides a more detailed delineation of tumor regions and integrates biological characteristics of the tumor microenvironment, thereby addressing the limitations of biopsy methods and offering more accurate information for tumor grading. Habitat imaging, an emerging tumor characterization technique, subdivides the tumor region into multiple “habitats” through clustering analysis of multimodal imaging data. Each habitat represents a tissue microenvironment with similar imaging features, offering a more accurate tumor characterization when combined with the biological characteristics of the tumor microenvironment [7–9]. Habitat features can be both visualized and quantified. For instance, calculating the volume fraction of each habitat enables assessment of the tumor’s composition. These features can serve as biomarkers to sensitively monitor dynamic changes in tumor composition, thereby aiding in the diagnosis of tumor malignancy, evaluation of therapeutic efficacy, and prediction of histopathological markers [10, 11]. While habitat imaging has been widely applied in the study of cancers such as hepatocellular carcinoma, lung cancer, and breast cancer [12–14], its application in renal tumors remains relatively limited. Habitat imaging, as a tumor biomarker, has not yet fully overcome the translational gaps defined in the “Imaging Biomarker Roadmap for Cancer Research“ [15]. However, with the development of this technology, it demonstrates significant potential for clinical applications. Nevertheless, the aim of this study is to explore the application of habitat imaging in clear cell renal cell carcinoma (ccRCC) and contribute to overcoming these gaps and advancing its clinical translation in the future.

Although triphasic contrast-enhanced CT is not commonly used in other types of renal diseases, its application in renal tumors such as clear cell renal cell carcinoma (ccRCC) and papillary renal cell carcinoma has proven to be of irreplaceable importance [16, 17]. By performing multi-phase contrast-enhanced imaging, triphasic CT provides detailed information on tumor perfusion, hemodynamics, and tumor boundary delineation, thereby offering a reliable foundation for assessing tumor heterogeneity. In contrast, non-contrast CT provides only static anatomical information and lacks the ability to capture dynamic perfusion changes, rendering it inadequate for assessing tumor heterogeneity. Given that habitat imaging depends on temporal contrast enhancement patterns to identify biologically distinct intratumoral subregions, this study exclusively utilized triphasic contrast-enhanced CT to enable a more accurate and comprehensive characterization of tumor habitats relevant to histopathological grading in ccRCC.

**Fig. 1:**
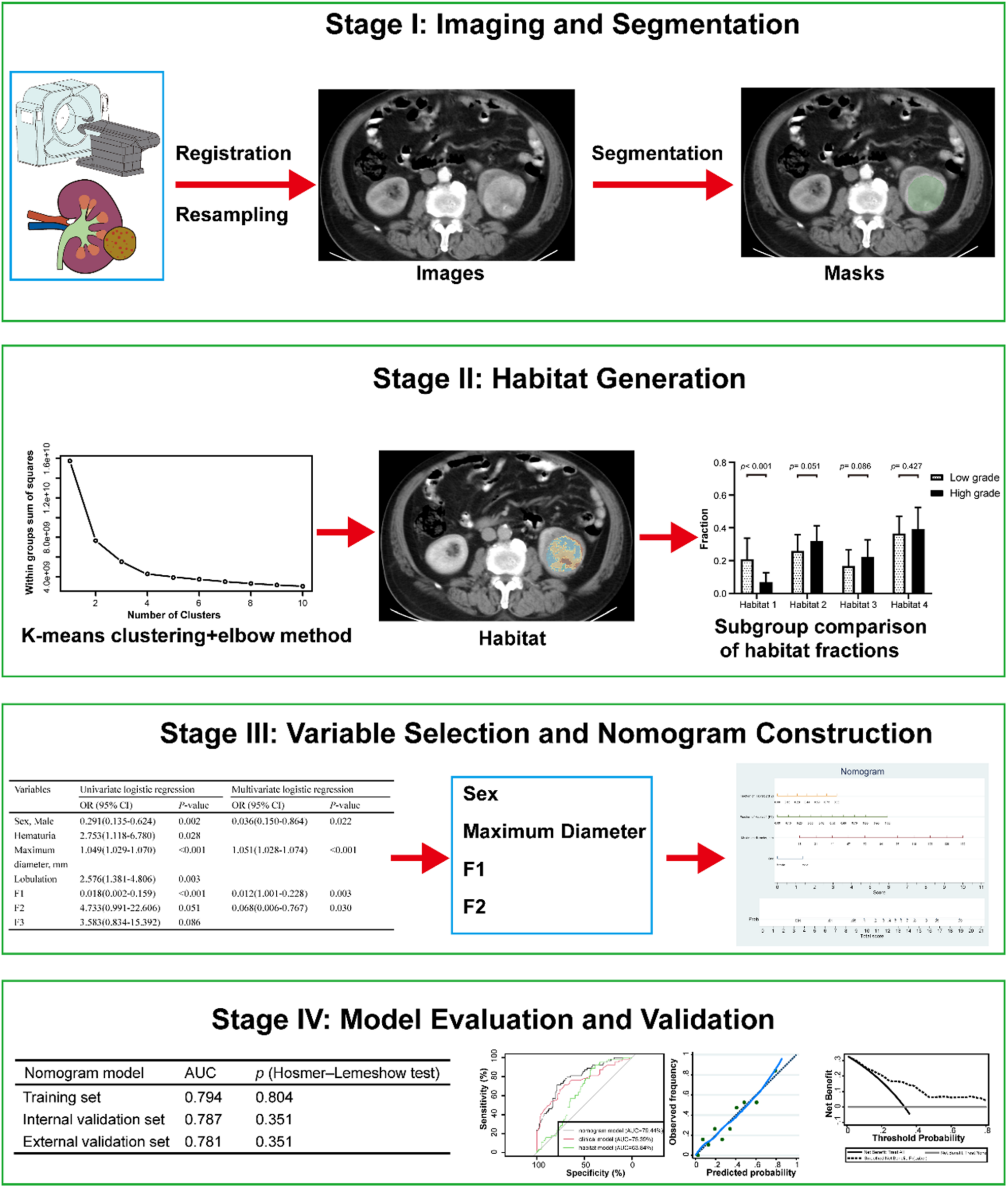
The technical flowchart of this study

Despite the promising potential of habitat imaging, its application in ccRCC remains limited, and previous studies have seldom integrated multi-phase CT-derived habitat features with clinical indicators for histological grade prediction. Moreover, most existing models rely on single-phase imaging or conventional radiomic features, which may inadequately capture the complex perfusion dynamics and microenvironmental heterogeneity of ccRCC. This limitation hampers the development of robust, non-invasive tools for accurate preoperative tumor grading. To address these gaps, the present study proposes an integrative approach that combines triphasic contrast-enhanced CT with habitat imaging to quantitatively evaluate the spatial distribution and fraction of distinct intratumoral habitats. By extracting and analyzing these imaging-based features alongside clinical parameters, we aim to develop a nomogram model capable of accurately predicting WHO/ISUP histological grades of ccRCC prior to surgery. The overall methodological framework is illustrated in Fig. 1.

## Materials and methods

### Patient information

**Fig. 2:**
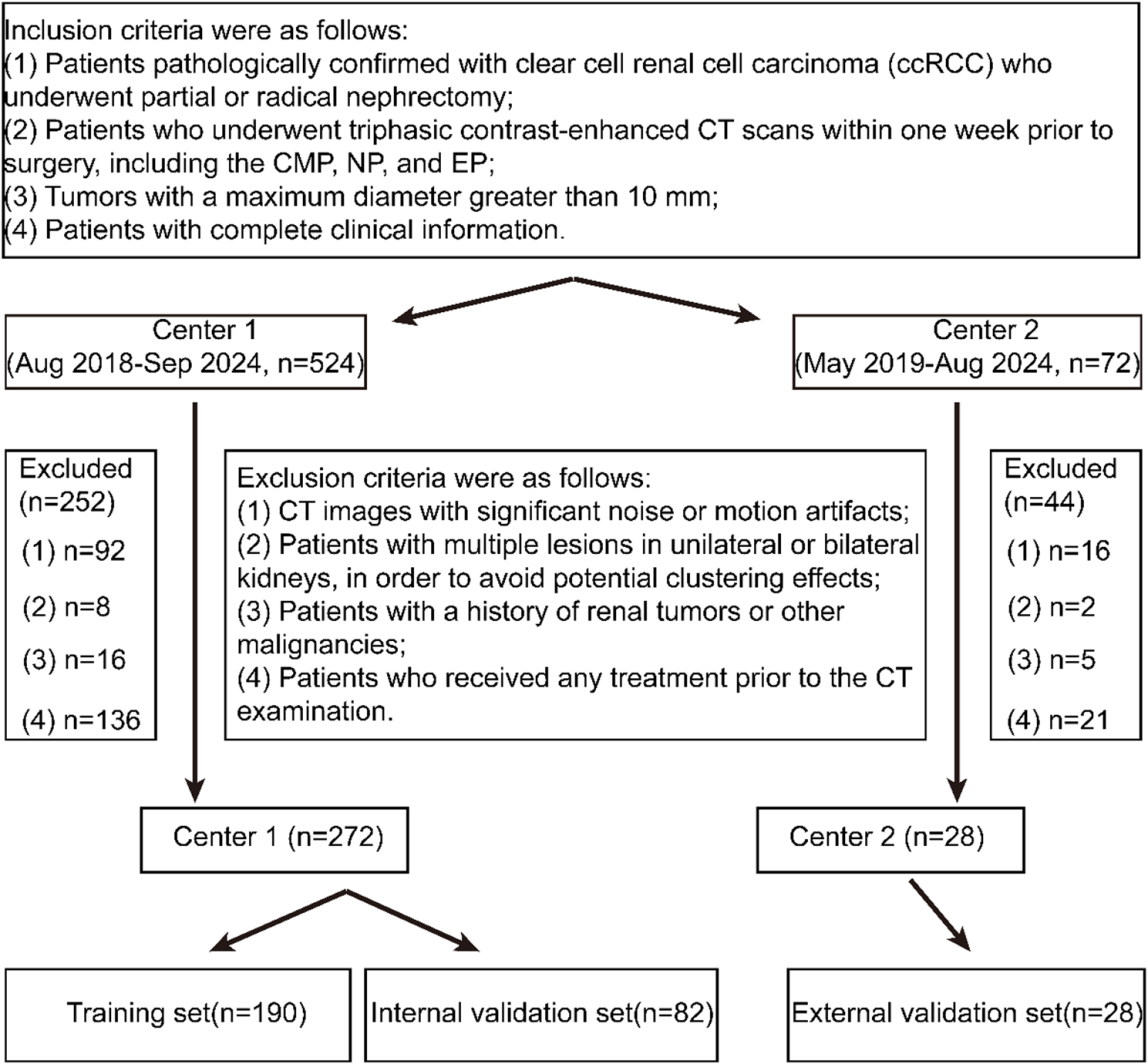
Enrollment flowchart of the study population

This retrospective study was approved by the hospital’s ethics committee, and the requirement for patient informed consent was waived. The study included patients diagnosed with renal tumors who underwent abdominal CT scans at Northern Jiangsu People’s Hospital (Center 1) in Jiangsu from August 2018 to September 2024, and at the Second People’s Hospital of Huai’an in Jiangsu (Center 2) from May 2019 to August 2024. All data were retrieved from the Picture Archiving and Communication System (PACS) at each institution. The inclusion Criteria were as follows: (1) Patients with pathological confirmation of clear cell renal cell carcinoma (ccRCC) who underwent partial or radical nephrectomy; (2) Patients who had undergone triphasic contrast-enhanced CT scans within one week prior to surgery, including the corticomedullary phase (CMP), nephrographic phase (NP), and excretory phase (EP); (3) Patients with a tumor maximum diameter greater than 10 mm; (4) Patients with complete clinical data. Exclusion Criteria for all participants were: (1) Patients whose CT images were affected by significant noise or artifacts; (2) Patients with multiple lesions in one or both kidneys, to avoid potential clustering effects; (3) Patients with a history of renal tumors or other malignancies; (4) Patients who had received treatment prior to the CT scan. According to previous studies, the WHO/ISUP grading system defines grades I–II as low-grade and grades III–IV as high-grade. A total of 272 patients from Center 1 were enrolled, including 88 with high-grade ccRCC and 184 with low-grade ccRCC. Patients were randomly assigned to the training set (*n* = 190) and internal validation set (*n* = 82) in a 7:3 ratio. Additionally, 28 patients from Center 2 were included as an external validation set, consisting of 11 high-grade and 17 low-grade ccRCC patients. The patient enrollment flowchart is shown in Fig. 2.

### CT scanning

CT scans were performed using a 64-slice spiral CT scanner (LightSpeed VCT CT99, GE, USA) and a gemstone spectral CT scanner (Discovery CT 750HD, GE, USA). All patients underwent scanning according to a standardized protocol. The scanning parameters were as follows: slice thickness and inter-slice spacing were both set to 5 mm, and the matrix size was 512 × 512. The window width was set to 250–450 Hounsfield units (HU), and the window level to 30–50 HU. A total of 70 mL of non-ionic contrast agent (iohexol) was intravenously injected into the forearm of each patient using a high-pressure injector at a rate of 3–5 mL/s. The delay times for the three-phase renal CT enhancement were as follows: CMP 25–30 s, NP 50–60 s, and EP 3–5 min.

### Image registration and resampling

All imaging data were preprocessed using 3D Slicer (version 5.6.2, https://www.slicer.org) for spatial alignment and standardization. Initially, image registration across different sequences or time points was performed using the General Registration (Advanced Normalization Tools [ANTs]) module. Rigid registration was applied to ensure consistency in spatial structure.

Subsequently, the Resample Scalar Volume module was used to resample the images to a uniform voxel size. The resampling resolution was set to 1.0 × 1.0 × 3.0 mm, with linear interpolation as the chosen method. This procedure aimed to minimize the impact of differences in scanning equipment and parameters, thereby enhancing the accuracy and comparability of subsequent analyses.

No ComBat or other intensity/feature harmonization was applied across scanners or institutions.

### Image segmentation

CT images were exported from the PACS and transferred to an independent workstation. Two experienced radiologists (Z.L. and S.N.) independently delineated the VOIs using 3D Slicer software, without knowledge of the pathological information. The VOIs were manually outlined on CMP images, and NP and EP images were used as references for verification, ensuring that the VOIs encompassed the entire tumor area of ccRCC. In cases of discrepancies, a radiologist with 20 years of experience (Y.J.) reviewed and adjusted the segmentation. The VOIs were drawn manually and subsequently mapped to the images from other phases for further analysis. Inter- and intra-observer reproducibility of VOI delineation was assessed using the intraclass correlation coefficient (ICC). The ICC values were ≥ 0.85, indicating good agreement between segmentations.

Tumor lobulation was evaluated on multiphasic CT images (with multiplanar review when needed) by the same two radiologists (Z.L. and S.N.) blinded to pathologic information. Lobulation was defined as a lobulated/scalloped tumor contour with ≥ 2 distinct outward convex protrusions separated by intervening indentations and was recorded as a binary variable (present/absent). Disagreements were resolved by consensus with a senior radiologist (Y.J., 20 years’ experience).

### Habitat mapping

In habitat mapping of ccRCC tumor regions, each voxel in the tumor area is associated with data from three phases of contrast-enhanced CT: CMP, NP, and EP. First, the CT values (in HU) of each voxel are extracted. These values, along with the three-dimensional parameters from all voxels across the entire patient cohort, are used to construct a data matrix of size *N* × 3, where N represents the total number of voxels in the image, and 3 corresponds to the CT values from the CMP, NP, and EP phases.

Subsequently, K-means clustering analysis is performed on these data to identify the habitat characteristics of the tumor regions across the different contrast-enhanced phases [18]. The elbow method is a widely used technique for determining the optimal number of clusters in K-means clustering. It is based on evaluating the sum of squared errors (*SSE*) for different values of *k*, which represents the number of clusters [19, 20]. The *SSE* is calculated as follows:

$$SSE\left( k \right) = \sum\nolimits_{i = 1}^N {{{\left\| {{x_i} - \mu {c_i}} \right\|}^2}} $$

where *N* is the total number of data points, *xi* denotes the *i*-th data point, *µ*_*c*_*i* is the centroid of the cluster to which *xi* belongs, and ‖*xi − µ*_*c*_*i*‖² is the squared Euclidean distance between the data point and its assigned cluster centroid.

As the number of clusters *k* increases, the *SSE* decreases. However, the marginal gain in clustering performance diminishes beyond a certain point. The “elbow point” is identified as the value of *k* at which the rate of *SSE* reduction sharply declines, indicating a balance between underfitting and overfitting. This point is considered the optimal number of clusters.

Finally, the volumetric fraction of each habitat in ccRCC is quantitatively determined using the following formula:

$$\eqalign{F\_\left\{ i \right\}\> = \>\backslash frac & \{ \backslash sum\backslash text\{ Number\>of\>Voxels\>in\>Habitat\} \_\{ i\} \cr& \backslash text\{ Within\>VOI\} \} \{ \backslash sum \cr& \backslash text\{ Number\>of\>All\>Voxels\>Within\>VOI\} \} \>\backslash \>times\>100\backslash \>\% \cr} $$

### Statistical analysis

All continuous variables were tested for normality using the Shapiro-Wilk test before statistical description. Variables with a normal distribution were presented as means ± standard deviations (SD), while non-normally distributed variables were expressed as medians with interquartile ranges (IQR). Categorical variables were summarized as counts and percentages. A significance level of *P* < 0.05 was used for descriptive statistical analysis. Inter- and intra-observer reproducibility of VOI delineation was evaluated using the intraclass correlation coefficient (ICC), with ICC values ≥ 0.85 indicating good agreement. Clinical characteristics and habitat volume scores were compared between low-grade and high-grade ccRCC groups using univariate logistic regression analysis. Variables with a *P*-value < 0.1 were retained for multivariate logistic regression to minimize the risk of excluding clinically relevant predictors, in accordance with established methodological recommendations [21, 22]. Stepwise backward selection was applied for variable selection, and a nomogram was subsequently constructed based on the selected clinical risk factors and habitat volume scores. Comparison of different models using the DeLong test, with a *P*-value less than 0.05 considered statistically significant. Model performance was evaluated using the area under the receiver operating characteristic curve (AUC), calibration curve, Hosmer–Lemeshow (HL) test, and decision curve analysis (DCA). Variable selection was performed using IBM SPSS (version 26.0), model construction and evaluation were conducted in STATA (version 15.0), and additional statistical analyses were carried out with R (version 4.0.2; https://www.r-project.org) and Python (version 3.7.2; https://www.python.org).

## Results

### Patient characteristics

This multicenter retrospective study included a total of 272 patients from Center 1, with a 7:3 random allocation ratio. Among these, 190 patients were assigned to the training set, and 82 patients to the internal validation set. The training set comprised 127 patients with low-grade tumors and 63 patients with high-grade tumors; the internal validation set included 57 low-grade and 25 high-grade patients. Additionally, Center 2 served as an independent external validation set, comprising 28 patients, including 17 with low-grade tumors and 11 with high-grade tumors. Detailed characteristics of patients stratified by tumor grade across the training, internal validation, and external validation sets are presented in the Table 1.

**Table 1:** Clinical and CT morphological characteristics of patients in the training and two validation sets

Variables	Training set(*n* = 190)			Internal validation set(*n* = 82)			External validation set(*n* = 28)		
	Low grade (*n* = 127)	High grade (*n* = 63)	*P*-value	Low grade (*n* = 57)	High grade (*n* = 25)	*P*-value	Low grade (*n* = 17)	High grade (*n* = 11)	*P*-value
Sex, Male (%)	77(60.6)	53(84.1)	0.002	37(64.9)	23(92.0)	0.020	12(70.6)	10(90.9)	0.225
Age, years	62.12$\:\pm\:$11.18	63.43$\:\pm\:$9.49	0.424	60.74$\:\pm\:12.28$	64.36$\:\pm\:9.89$	0.197	61.82$\:\pm\:12.40$	64.09$\:\pm\:12.78$	0.631
Hypertension (%)	65(51.2)	30(47.6)	0.644	28(49.1)	12(48.0)	0.925	12(70.6)	7(63.6)	0.701
Diabetes (%)	21(16.5)	9(14.3)	0.689	9(15.8)	2(8.0)	0.350	6(35.3)	1(9.1)	0.145
Smoking (%)	29(22.8)	17(27.0)	0.530	15(26.3)	9(36.0)	0.377	4(23.5)	2(18.2)	0.737
Drinking (%)	11(8.7)	6(9.5)	0.845	10(17.5)	8(32.0)	0.151	5(29.4)	1(9.1)	0.225
Backache (%)	27(21.3)	14(22.2)	0.879	15(26.3)	6(24.0)	0.825	2(11.8)	1(9.1)	0.824
Hematuria (%)	10(7.9)	12(19.0)	0.028	5(8.8)	6(24.0)	0.073	1(5.9)	4(36.4)	0.067
Location, left (%)	59(46.5)	37(58.7)	0.112	33(57.9)	12(48.0)	0.408	9(52.9)	5(45.5)	0.699
Maximum diameter, mm	43.46$\:\pm\:14.22$	60.98$\:\pm\:26.35$	< 0.001	46.42$\:\pm\:20.92$	64.92$\:\pm\:23.59$	0.002	31.59$\:\pm\:12.20$	48.73$\:\pm\:25.11$	0.050
Arteriovenous thrombosis (%)	5(3.9)	4(6.3)	0.465	1(1.8)	2(8.0)	0.205	2(11.8)	4(36.4)	0.137
Lymphadenopathy (%)	16(12.6)	13(20.6)	0.151	7(12.3)	5(20.0)	0.367	3(17.6)	4(36.4)	0.272
Cystic degeneration (%)	63(49.6)	29(46.0)	0.643	20(35.1)	9(36.0)	0.937	7(41.2)	5(45.5)	0.823
Necrosis (%)	93(73.2)	48(76.2)	0.661	40(70.2)	21(84.0)	0.194	14(82.4)	10(90.9)	0.534
Lobulation (%)	38(29.9)	33(52.4)	0.003	21(36.8)	16(64.0)	0.026	2(11.8)	5(45.5)	0.058

Univariate logistic regression analysis revealed significant differences between low-grade and high-grade ccRCC patients in the training set regarding sex, hematuria, maximum diameter, and tumor lobulation (*P* < 0.05). Similarly, in the internal validation set, sex, maximum diameter, and tumor lobulation also exhibited significant differences (*P* < 0.05). In the external validation set, a statistically significant difference in maximum diameter was observed (*P* < 0.05).

### Habitat classification and subgroup comparison

**Fig. 3:**
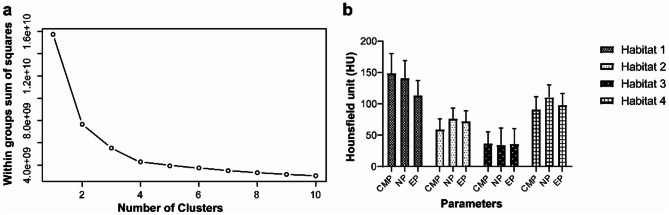
Habitat encoding and characteristics. (**a**) The relationship between different clustering numbers (k) and the SSE, indicating the optimal habitat encoding. (**b**) Quantitative characteristics of the four habitats

**Fig. 4:**
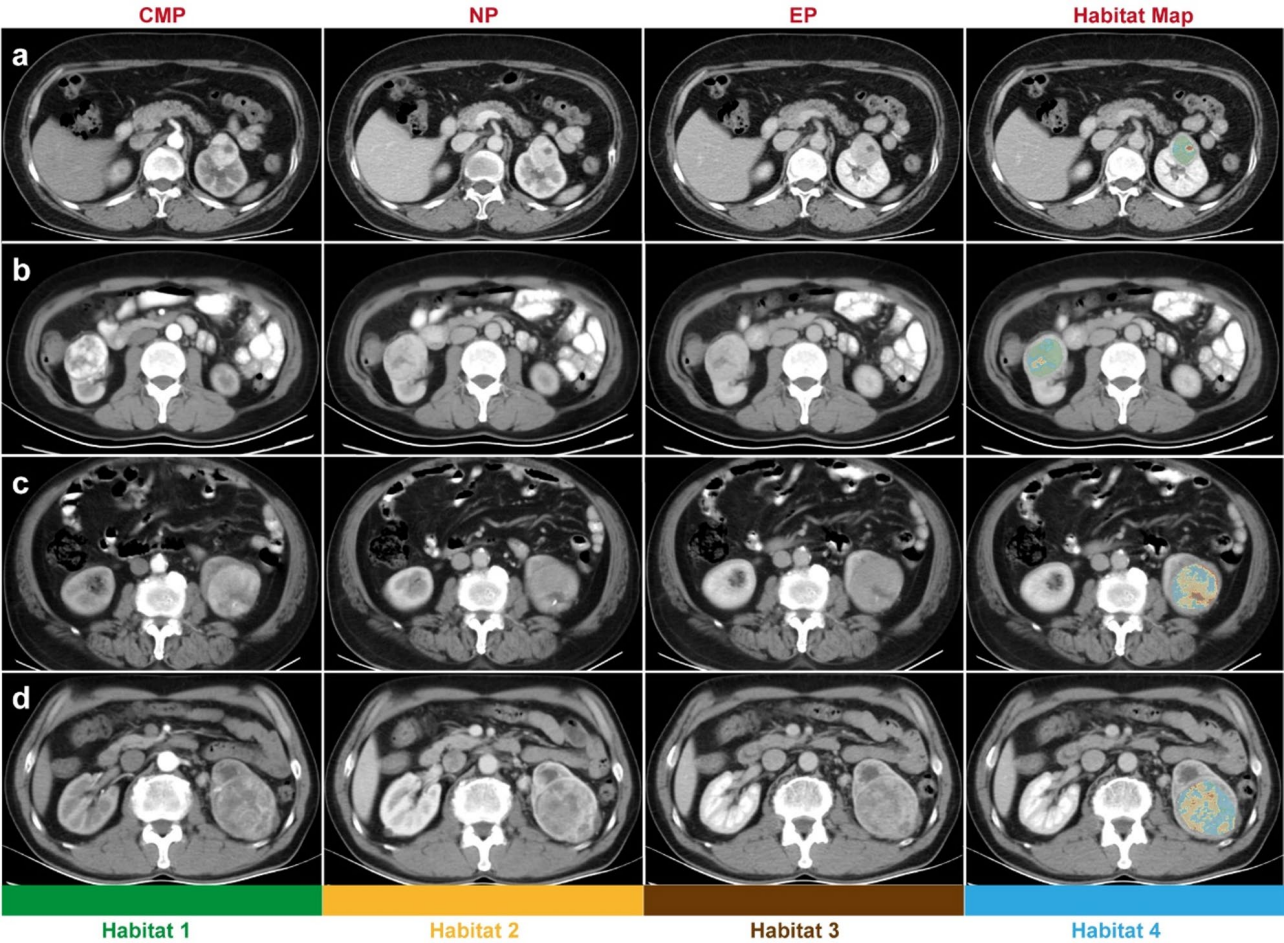
Representative CT images of two low-grade ccRCCs (**a**, **b**) and two high-grade ccRCCs (**c**, **d**). Note: (1) Tumor habitat maps overlaid on EP-phase images. (2) Each color represents a distinct tumor habitat

To assess clustering performance across different numbers of clusters (*k*), we plotted the variation of the sum of *SSE* for each *k* value. When *k* = 4, the *SSE* curve displayed a clear “elbow” point (Fig. 3a), suggesting that partitioning the data into four clusters achieves an optimal balance between intra-cluster tightness and model complexity. The representative characteristics of each Habitat are provided in Fig. 3b: Habitat 1: CMP = 148.776 ± 31.368 HU, NP = 141.015 ± 27.948 HU, EP = 113.560 ± 23.338 HU; Habitat 2: CMP = 58.965 ± 17.397 HU, NP = 76.482 ± 16.554 HU, EP = 72.128 ± 17.023 HU; Habitat 3: CMP = 36.851 ± 18.669 HU, NP = 34.450 ± 27.000 HU, EP = 35.833 ± 24.619 HU; Habitat 4: CMP = 90.916 ± 20.907 HU, NP = 110.258 ± 20.344 HU, EP = 97.906 ± 18.737 HU(CT values represent the mean ± standard deviation for each habitat). Representative CT images are shown in Fig. 4.

**Fig. 5:**
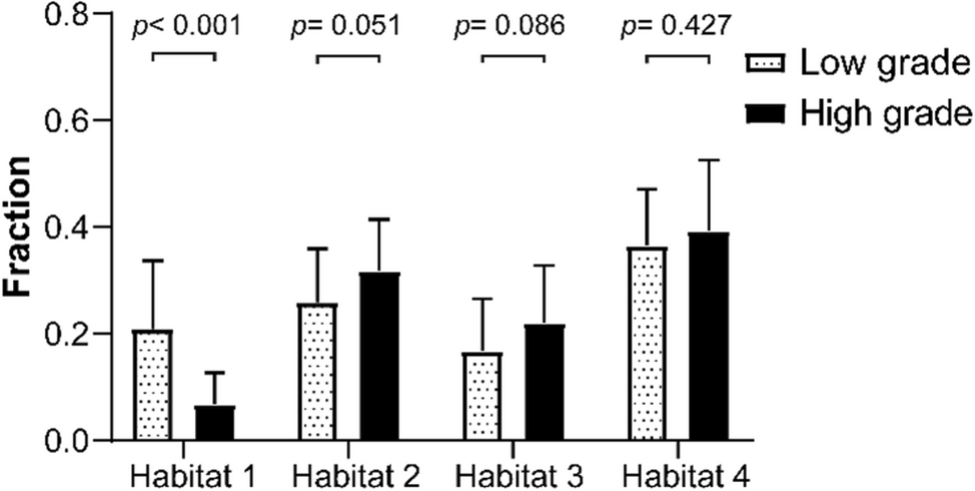
Subgroup comparison of habitat fractions between low-grade and high-grade ccRCC

Moreover, an analysis of the training dataset revealed that, compared with low-grade clear cell renal cell carcinoma (ccRCC), high-grade ccRCC exhibited a significantly lower fraction of Habitat 1 (F1) (F1: 0.068 ± 0.110 vs. 0.209 ± 0.255, *P* < 0.001). No significant difference was observed in the fraction of Habitat 2 (F2), Habitat 3 (F3) and Habitat 4(F4) between high-grade and low-grade ccRCC (F2: 0.317 ± 0.193 vs. 0.259 ± 0.191, *P* = 0.051; F3: 0.221 ± 0.212 vs. 0.167 ± 0.196, *P* = 0.086; F4: 0.393 ± 0.266 vs. 0.365 ± 0.213, *P* = 0.427) (Fig. 5).

### Risk factors and nomogram construction

**Fig. 6:**
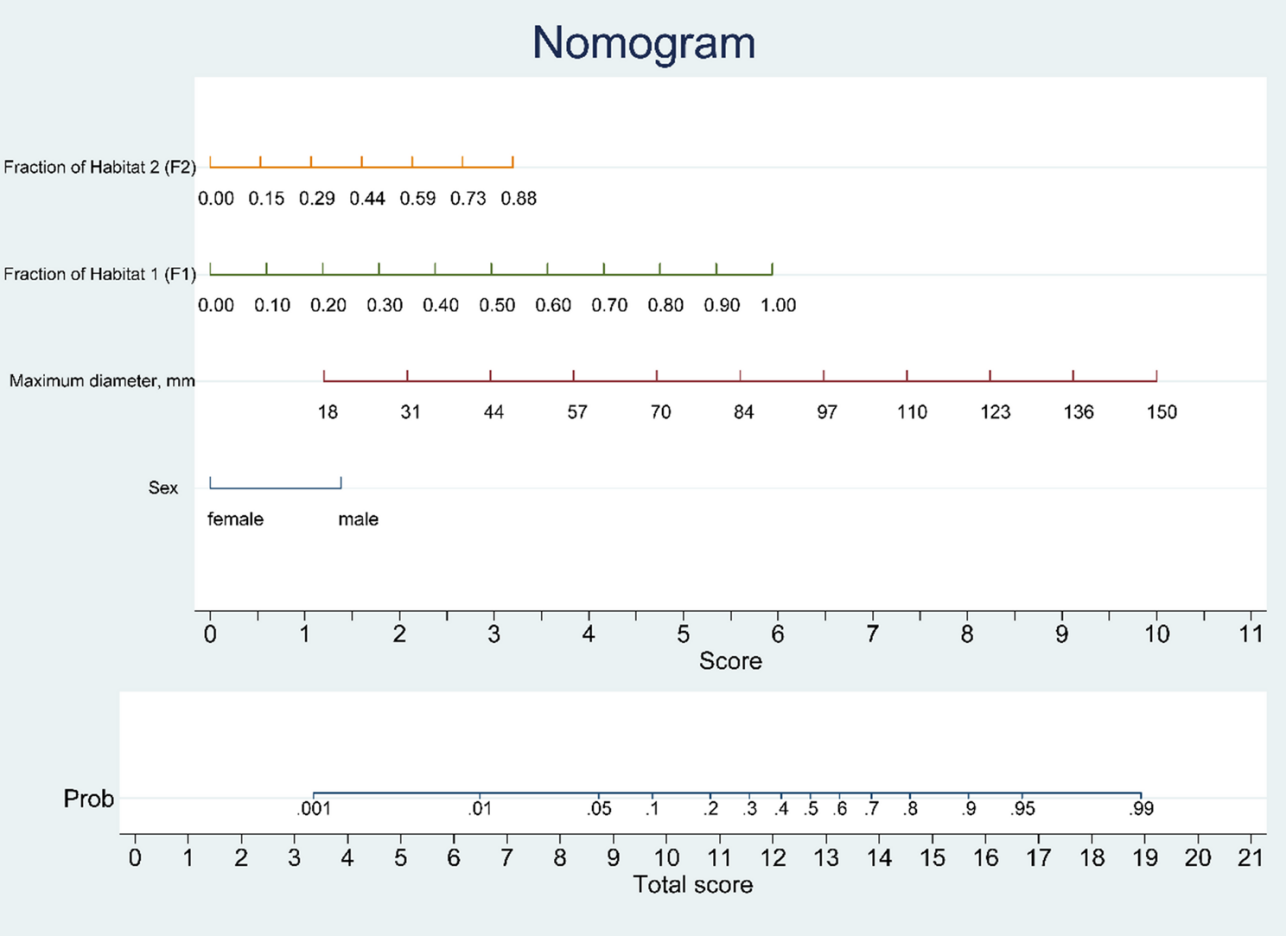
Nomogram model illustration

In the training cohort, univariate logistic regression was initially performed to assess the clinical variables, including Sex, Hematuria, Maximum Diameter, and Lobulation (with a significance threshold of *P* < 0.1). These variables were subsequently incorporated into the multivariate logistic regression model, along with habitat-derived features F1, F2, and F3 (*P* < 0.1). Variable selection was conducted using the stepwise backward logistic regression (LR) approach. Ultimately, Sex, Maximum Diameter, F1, and F2 were identified as independent risk factors for the WHO/ISUP grading of ccRCC (Table 2). These four variables were then used to construct the nomogram model (Fig. 6).

**Table 2:** Univariate and multivariate logistic regression analysis of clinical and habitat risk factors

Variables	Univariate logistic regression		Multivariate logistic regression	
	OR (95% CI)	*P*-value	OR (95% CI)	*P*-value
Sex, Male	0.291(0.135–0.624)	0.002	0.036(0.150–0.864)	0.022
Hematuria	2.753(1.118–6.780)	0.028		
Maximum diameter, mm	1.049(1.029–1.070)	< 0.001	1.051(1.028–1.074)	< 0.001
Lobulation	2.576(1.381–4.806)	0.003		
F1	0.018(0.002–0.159)	< 0.001	0.012(1.001–0.228)	0.003
F2	4.733(0.991–22.606)	0.051	0.068(0.006–0.767)	0.030
F3	3.583(0.834–15.392)	0.086		

**F1–3**: Habitat volume proportion in the tumor (as a decimal)

### Model evaluation and validation

**Fig. 7:**
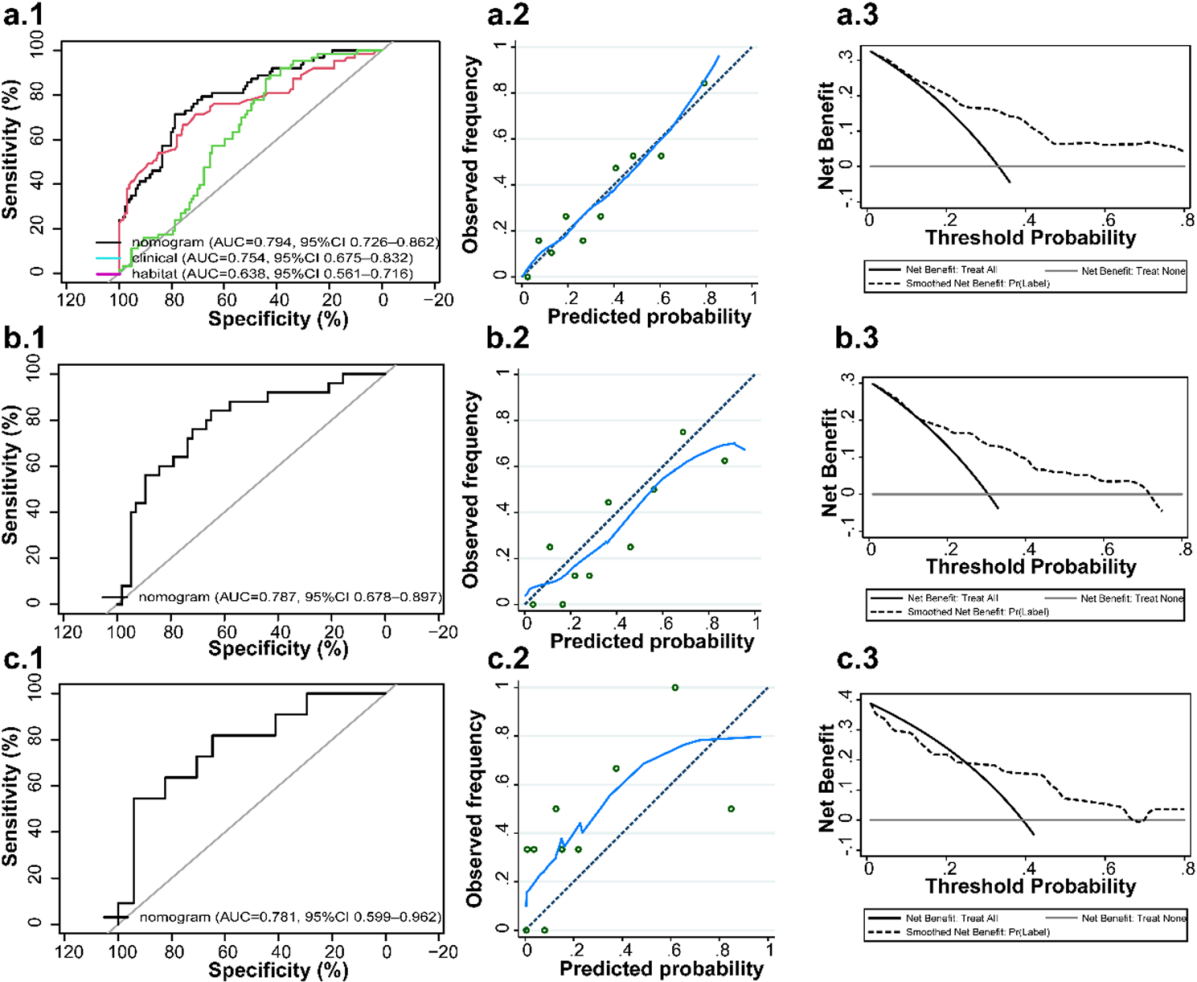
Model evaluation and validation. (**a.1**) ROC curves of the nomogram model, clinical model, and habitat model in the training set. (**a.2**-**a.3**) Calibration curve, and decision curve of the nomogram model in the training set. (**b**.1-**b.3**) ROC curve, calibration curve, and decision curve of the nomogram in the internal validation set. (**c.1**-**c.3**) ROC curve, calibration curve, and decision curve of the nomogram in the external validation set

Furthermore, we established a clinical model based on Sex and Maximum Diameter, and a habitat model based on F1 and F2. Discriminatory performance of the nomogram model, clinical model, and habitat model was analyzed separately in the training set (Fig. 7a.1). The results demonstrated that the nomogram model exhibited higher discriminatory power, and the DeLong test (*P* = 0.047) showed a statistically significant difference between the nomogram and clinical models. The nomogram model was then further evaluated and validated.

The nomogram model demonstrated excellent predictive performance across the training, internal validation, and external validation sets. In the training set, the model yielded an AUC of 0.794 (95% CI: 0.726–0.862). Calibration curves indicated strong agreement between predicted probabilities and actual outcomes (Hosmer–Lemeshow test, *P* = 0.804). Decision curve analysis (DCA) revealed substantial net benefit across a wide range of risk thresholds (Fig. 7a.1-a.3).

In the internal validation set, the model maintained robust discriminatory power (AUC = 0.787, 95% CI: 0.678–0.897). Calibration analysis confirmed satisfactory consistency between predicted and observed outcomes (Hosmer–Lemeshow test, *P* = 0.351), with DCA showing comparable clinical benefit to the training set (Fig. 7b.1-b.3).

In the external validation set, the nomogram exhibited stable predictive accuracy (AUC = 0.781, 95% CI: 0.599–0.962). Although slight overprediction was observed at higher risk levels, the calibration curve showed acceptable consistency overall (Hosmer–Lemeshow test, *P* = 0.351). DCA results reaffirmed the model’s clinical utility, supporting the reproducibility of model performance in an independent cohort, although the estimate is imprecise due to the small sample size (Fig. 7c.1-c.3).

### Comparison of the nomogram model with the R.E.N.A.L score model

**Fig. 8:**
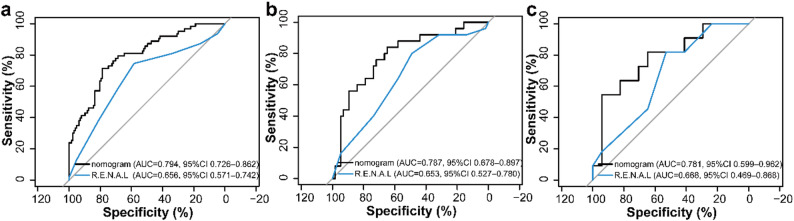
ROC curves comparing the nomogram and the total R.E.N.A.L nephrometry score model in the training (**a**), internal-validation (**b**), and external-validation (**c**) cohorts. AUCs (95% CIs) are shown in each panel

To contextualize model performance against a commonly used preoperative anatomic scoring tool [23], we compared the nomogram with a logistic regression model based on the total R.E.N.A.L nephrometry score (Radius, Exophytic/endophytic, Nearness, Anterior/posterior, Location). The distribution of R.E.N.A.L components and the total score by WHO/ISUP grade is summarized in Supplementary Table S1. The nomogram yielded AUCs of 0.794, 0.787, and 0.781 across the training, internal-validation, and external-validation cohorts, respectively, compared with 0.656, 0.653, and 0.668 for the total R.E.N.A.L score model (Table 3; Fig. 8). AUC values with their corresponding 95% confidence intervals are provided in Table 3; Fig. 8. The AUC difference favored the nomogram in the training and internal-validation cohorts (DeLong *P* = 0.001 and 0.041), while the difference was not statistically significant in the external-validation cohort (DeLong *P* = 0.330), likely due to limited power in the small external sample.

**Table 3:** Discriminative performance of the nomogram versus the total R.E.N.A.L nephrometry score for preoperative WHO/ISUP grade stratification in clear cell renal cell carcinoma

Model	Training AUC (95% CI)	Internal validation AUC (95% CI)	External validation AUC (95% CI)	DeLong *P* vs. Nomogram (Training)	DeLong *P* vs. Nomogram (Internal)	DeLong *P* vs. Nomogram (External)
Nomogram	0.794 (95% CI: 0.726–0.862)	0.787, (95% CI: 0.678–0.897)	0.781, (95% CI: 0.599–0.962)	–	–	–
Total R.E.N.A.L scores	0.656 (95% CI: 0.571–0.742)	0.653 (95% CI: 0.527–0.780)	0.668 (95% CI: 0.469–0.868)	0.001	0.041	0.330

AUC 95% CIs were estimated using the DeLong method. The total R.E.N.A.L score was calculated as R + E+N + L, with A and H recorded as descriptors and not included in the numeric total

## Discussion

This study introduces a novel habitat imaging approach based on triphasic contrast-enhanced computed tomography (CT) to predict WHO/ISUP grade in clear cell renal cell carcinoma (ccRCC) prior to surgery. CT images acquired during the corticomedullary (CMP), nephrographic (NP), and excretory (EP) phases were used to perform clustering analysis and identify intratumoral regions with distinct perfusion patterns. Habitat 1, defined by high CMP enhancement with progressive decline in NP and EP, and Habitat 2, characterized by low CMP, elevated NP, and decreased EP enhancement, showed an inverse distribution between low-grade and high-grade ccRCC. Both habitats were identified as independent predictors in multivariate logistic regression. A nomogram combining habitat fractions, sex, and tumor size demonstrated promising performance in both training and multicenter validation cohorts.

Triphasic contrast-enhanced CT is widely accessible in clinical practice and provides reproducible, time-resolved data that reflect tumor vascularity and microenvironment [24, 25]. In this study, habitat clustering effectively captured the spatial heterogeneity of ccRCC, and the enhancement characteristics of key habitats provided incremental value for tumor grading. This noninvasive, imaging-based “virtual biopsy [26, 27]” method may overcome the sampling limitations of needle biopsy and support accurate preoperative grading and individualized treatment planning.

In prior literature, noninvasive preoperative grading models for ccRCC have predominantly relied on CT radiomics and MRI-based approaches [28–30]. While these methods can achieve promising discrimination, they often face challenges in reproducibility and generalizability across centers. In contrast, our triphasic CT habitat model leverages routinely acquired multiphasic CT data and a low-dimensional set of enhancement-defined intratumoral habitats, which may facilitate integration into routine clinical workflows. By combining interpretable habitat fractions—reflecting contrast uptake and washout heterogeneity—with simple clinical variables, the proposed nomogram may offer competitive performance while being easier to implement and more accessible than complex radiomics approaches.

Based on the elbow method, ccRCC was categorized into four distinct habitats, and habitat fractions were associated with WHO/ISUP grade. Specifically, the fraction of Habitat 1 was lower in high-grade than in low-grade tumors (*P* = 0.003), whereas the fraction of Habitat 2 was higher in high-grade tumors (*P* = 0.030). Habitat imaging leverages intratumoral subregions defined by distinct contrast uptake and washout kinetics, which may serve as imaging surrogates of heterogeneous microenvironments (e.g., perfusion, permeability, and hypoxia-related states) [31]. Within this framework, Habitat 1—showing strong corticomedullary enhancement with subsequent washout—may reflect relatively well-perfused viable components with preserved functional vascular supply and, indirectly, higher cellular viability, as early enhancement is more commonly observed in viable tumor regions where microvascular delivery remains intact [32]. In contrast, Habitat 2—showing low early enhancement with relatively delayed nephrographic enhancement and reduced excretory enhancement—may indicate hypoperfused regions with heterogeneous or immature microvasculature, potentially reflecting angiogenic remodeling and uneven microvessel distribution that are frequently observed in aggressive ccRCC [33]. This enhancement pattern may also capture less viable components such as necrosis or mixed low-attenuation areas, which tend to occur more often in higher-grade disease and contribute to intratumoral heterogeneity [34]. Importantly, these habitats represent imaging-defined phenotypes rather than direct histopathologic labels; voxel-wise pathologic or molecular correlation (e.g., necrosis quantification, microvessel density, or angiogenesis/hypoxia markers) was not available in this retrospective cohort. Therefore, the proposed biological interpretations should be considered hypothesis-generating and warrant prospective validation using targeted histology and/or molecular markers.

Additionally, multivariate logistic regression analysis identified sex and maximum tumor diameter as independent clinical risk factors. Specifically, male patients had significantly higher tumor grades than female patients (*P* = 0.022), and tumor size was significantly positively correlated with tumor grade (*P* < 0.001). These findings are in agreement with previous studies [35, 36], which emphasize the close association between sex, tumor size, and the pathological grading of ccRCC. Subsequently, a nomogram model, clinical model, and habitat model were constructed by integrating clinical variables and habitat fractions. The nomogram model exhibited the highest discriminatory ability, and DeLong test (*P* = 0.047) showed a statistically significant difference between the nomogram and clinical models, indicating that the imaging features of subregions provided, to some extent, incremental value to the nomogram’s discriminatory power. These features primarily reflect the heterogeneity of the disease and offer additional information regarding distinct pathological characteristics. The nomogram model demonstrated excellent diagnostic performance, with good consistency between predicted and actual values. Furthermore, the model’s high net benefit in both internal and external validation sets further confirmed its stability and reliability.

To provide clinical context, when compared with the total R.E.N.A.L. nephrometry score—an established preoperative anatomic scoring system [23] —the nomogram showed higher discrimination in the training and internal-validation cohorts. This is clinically plausible because nephrometry scores primarily capture anatomic complexity, whereas the proposed nomogram additionally incorporates enhancement-defined intratumoral habitats, which may reflect heterogeneity in perfusion and microvascular function that is not represented by anatomy alone. Notably, the AUC difference was not statistically significant in the external-validation cohort and should be interpreted cautiously given the limited sample size and wide confidence intervals, underscoring the need for further validation in larger independent cohorts.

Although the proposed triphasic contrast-enhanced CT-based habitat imaging method demonstrates promising performance for preoperative grading of clear cell renal cell carcinoma (ccRCC), several limitations should be acknowledged. First, the external validation cohort was relatively small (*n* = 28), resulting in a wide AUC confidence interval and limited precision for out-of-sample performance; therefore, the generalizability of the model should be interpreted cautiously and requires confirmation in larger, prospectively collected multicenter cohorts. Second, selection bias may exist in the surgical patient cohort, as larger tumors are more likely to be high-grade ccRCC. To further assess the model’s broader applicability, future research should consider including patients with small tumors who undergo local treatments or active surveillance. Third, tumor VOIs were manually delineated, and inter- and intra-observer reproducibility was assessed using the inter-class correlation coefficient (ICC), with values ≥ 0.85 indicating good agreement. However, manual segmentation still has limitations. As habitat fractions were derived from the VOI, variability in segmentation may affect habitat quantification and model performance. Future studies should incorporate reproducibility testing across multiple readers and centers and explore automated or semi-automated segmentation methods to improve robustness and scalability. Fourth, although standardized preprocessing (HU-based quantification, cross-phase registration, and resampling) was performed, residual inter-scanner/institution variability may persist, potentially affecting clustering stability and generalizability. Future studies should explore feature-level harmonization (e.g., ComBat) or account for site/scanner effects while preserving enhancement kinetics. Fifth, we used a binary grading endpoint (WHO/ISUP I–II vs. III–IV) to provide a pragmatic low- versus high-grade stratification and to maintain adequate sample sizes for model training and validation. However, this dichotomization does not address the clinically challenging boundary between Grade 2 and Grade 3, where treatment decisions are often most nuanced. Therefore, the applicability of the proposed model for distinguishing Grade 2 from Grade 3 remains uncertain and warrants evaluation in larger cohorts using ordinal or multiclass modeling strategies. Moreover, the current habitat model was constructed solely based on triphasic CT imaging, without integrating functional imaging modalities such as diffusion-weighted imaging (DWI) or dynamic contrast-enhanced MRI (DCE-MRI). This limitation may hinder comprehensive characterization of tumor microstructure, diffusion properties, and hemodynamics. Future efforts should explore multimodal imaging fusion strategies to improve the accuracy of tumor phenotyping. Additionally, although four enhancement-based habitat subregions were defined, their underlying biological basis and pathological correlates remain to be further elucidated.

## Conclusion

In conclusion, the nomogram developed in this study offers a novel, non-invasive approach to preoperative prediction of WHO/ISUP grade in ccRCC by integrating triphasic CT imaging and clinical features. This tool has the potential to improve the accuracy of grading, aid in risk stratification, and guide personalized treatment planning, ultimately contributing to better patient management and outcomes. The clinical utility of this nomogram could be particularly valuable in settings where biopsy is not feasible or where there is diagnostic uncertainty, providing clinicians with a reliable alternative to invasive procedures.

## Supplementary Information

Below is the link to the electronic supplementary material.

Supplementary Material 1

## Data Availability

The data supporting the findings of this study are available from the corresponding author upon reasonable request. All relevant data are included in the article. Due to privacy or ethical restrictions, certain data may not be publicly available.

## Abbreviations

ccRCC: clear cell renal cell carcinoma
CMP: Corticomedullary phase
NP: Nephrographic phase
EP: Excretory phase
VOIs: Volumes of interest
ROC: Receiver operating characteristic
AUC: Area under the curve
HL: Hosmer-Lemeshow
DCA: Decision curve analysis
WHO/ISUP: World Health Organization/International Society of Urological Pathology
PACS: Picture Archiving and Communication System
ANTs: Advanced Normalization Tools
HU: Hounsfield units
VOIs: Volumes of interest
SSE: Sum of squared error
F1: Fraction of Habitat 1
F2: Fraction of Habitat 2
F3: Fraction of Habitat 3
F4: Fraction of Habitat 4
DWI: Diffusion-weighted imaging
DCE-MRI: Dynamic contrast-enhanced MRI
R.E.N.A.L: Radius, Exophytic/endophytic, Nearness, Anterior/posterior, Location
ICC: Inter-class correlation coefficient
