# Supplementary material for: A multicenter study on preoperative WHO/ISUP grading of clear cell renal cell carcinoma using triphasic contrast-enhanced CT-based habitat imaging
Source: BMC Med Imaging. 2026 Feb 27;26:174. doi: 10.1186/s12880-026-02236-z (PMC13045149; doi:10.1186/s12880-026-02236-z)
Supplement: Supplementary file 1 — Supplementary Material 1 [file 12880_2026_2236_MOESM1_ESM.docx]

**Table S1.** Distribution of R.E.N.A.L nephrometry score components and total score stratified by WHO/ISUP grade across cohorts.

| Variables | | Training set(n=190) | | *P*-value | Internal validation set(n=82) | | *P*-value | External validation set(n=28) | | *P*-value |
| --- | --- | --- | --- | --- | --- | --- | --- | --- | --- | --- |
|  |  | Low grade  (n=127) | High grade  (n=63) |  | Low grade  (n=57) | High grade  (n=25) |  | Low grade  (n=17) | High grade  (n=11) |  |
| R scores, n (%) | 1 | 62(48.8) | 14(22.2) | <0.001 | 25(43.9) | 4(16.0) | 0.003 | 15(88.2) | 1(9.1) | 0.010 |
|  | 2 | 58(45.7) | 25(39.7) |  | 26(45.6) | 10(40.0) |  | 2(11.8) | 7(63.6) |  |
|  | 3 | 7(5.5) | 24(38.1) |  | 6(10.5) | 11(44.0) |  | 0(0) | 3(27.3) |  |
| E scores, n (%) | 1 | 57(44.9) | 27(42.9) | 0.885 | 22(38.6) | 15(60.0) | 0.166 | 7(41.2) | 3(27.3) | 0.734 |
|  | 2 | 55(43,3) | 27(42.9) |  | 27(47.4) | 9(36.0) |  | 8(47.1) | 6(54.5) |  |
|  | 3 | 15(11.8) | 9(14.3) |  | 8(14.0) | 1(4.0) |  | 2(11.8) | 2(18.2) |  |
| N scores, n (%) | 1 | 45(35.4) | 12(19.0) | 0.028 | 18(31.6) | 3(12.0) | 0.190 | 11(64.7) | 0(0) | 0.069 |
|  | 2 | 32(25.2) | 14(22.2) |  | 12(21.1) | 6(24.0) |  | 5(29.4) | 2(18.2) |  |
|  | 3 | 50(39.4) | 37(58.7) |  | 27(47.4) | 16(64.0) |  | 1(5.9) | 9(81.8) |  |
| A scores, n (%) | A | 51(40.2) | 27(42.9) | 0.772 | 25(43.9) | 11(44.0) | 0.991 | 9(52.9) | 7(63.6) | 0.577 |
|  | P | 76(59.8) | 36(57.1) |  | 32(56.1) | 14(56.0) |  | 8(47.1) | 4(36.4) |  |
| L scores, n (%) | 1 | 34(26.8) | 13(20.6) | 0.554 | 17(29.8) | 2(8.0) | 0.134 | 5(29.4) | 3(27.3) | 0.975 |
|  | 2 | 40(31.5) | 24(38.1) |  | 12(21.1) | 7(28.0) |  | 7(41.2) | 5(45.5) |  |
|  | 3 | 53(41.7) | 26(41.3) |  | 28(49.1) | 16(64.0) |  | 5(29.4) | 3(27.3) |  |
| H, n (%) | No | 115(90.6) | 54(85.7) | 0.320 | 50(87.7) | 20(80.0) | 0.367 | 12(70.6) | 10(90.9) | 0.225 |
|  | Yes | 12(9.4) | 9(14.3) |  | 7(12.3) | 5(20.0) |  | 5(29.4) | 1(9.1) |  |
| Total R.E.N.A.L scores^1,2^ | | 7.43$\pm$1.88 | 8.48$\pm$2.06 | 0.001 | 7.77$\pm$1.94 | 8.8$\pm$1.80 | 0.031 | 6.24$\pm$1.44 | 8.91$\pm$1.04 | 0.007 |

R, radius; E, exophytic/endophytic properties; N, nearness of the tumor to the collecting system or sinus; A, anterior/posterior; L, location relative to the polar lines; H, renal hilar invasion

1 MD ± SD

2 Total R.E.N.A.L score was calculated as the sum of the numeric components R + E + N + L (range 4–12). The A (anterior/posterior) and H (hilar involvement) descriptors were recorded as categorical suffixes and were not included in the numeric total.
